# RNA Therapeutics in Viral Infections and Cancer: Mechanisms, Challenges, and Prospects: A Review

**DOI:** 10.3390/pharmaceutics18040431

**Published:** 2026-03-31

**Authors:** Evgenii Generalov, Alexei Shevelev, Dmitry Romanov, Olga Tarasova, Natalia Pozdniakova

**Affiliations:** 1 Faculty of Physics, Lomonosov Moscow State University, 119991 Moscow, Russia; 2 Vavilov Institute of General Genetics of Russian Academy of Sciences, Gubkina 3, 119991 Moscow, Russia; shevelab@vigg.ru; 3 Faculty of Biochemistry, Escola Normal e Ginasio Madre Teresa Michel, Joaquim Nabuco Street, 1015 Michel, Criciuma 88803-000, SC, Brazil; 4 Institute of Biomedical Chemistry of Russian Academy of Sciences, Pogodinskaya 10, 8, 119121 Moscow, Russia; olga.a.tarasova@gmail.com; 5 Blokhin National Medical Research Center of Oncology, Ministry of Health of the Russian Federation, Kashirskoe Shosse 24, 115522 Moscow, Russia; natpo2002@mail.ru

**Keywords:** siRNA, miRNA, mRNA, HIV, HCV, HBV, COVID-19, RNA therapeutics

## Abstract

**Background**: RNA therapeutics represent a rapidly advancing field with significant potential for treating viral infections and cancer. This review examines the current landscape of RNA-based strategies, including siRNA, miRNA mimics, and antisense oligonucleotides. For viral infections, the focus is on hepatitis B (HBV) and C (HCV), HIV, and SARS-CoV-2. Approaches include targeting viral transcripts directly (e.g., siRNAs against HBV surface antigen) or host factors critical for viral replication (e.g., anti-miR-122 miravirsen for HCV). The successful development of mRNA vaccines for COVID-19 is highlighted as a major breakthrough, demonstrating the feasibility of rapid RNA vaccine deployment. The manuscript reviews several RNA therapeutics in oncology that have reached clinical trials. These include TargomiR (a miR-16 mimic for mesothelioma), cobomarsen (an anti-miR-155 for lymphomas), and MRX34 (a miR-34a mimic for various solid tumours). The review also covers emerging candidates like an miR-221 inhibitor and various strategies for breast cancer, such as targeting Bcl-2, KRAS, and specific miRNAs. A critical challenge across both fields is developing efficient and safe delivery systems, including lipid nanoparticles, GalNAc conjugates, and bacterial minicells. Despite promising preclinical results, clinical translation has been hampered by issues like insufficient delivery efficiency to human tumours, toxicity, and the complex, interconnected regulatory networks of miRNAs, which can lead to unpredictable off-target effects. **Conclusions**: While RNA therapeutics hold immense promise, overcoming delivery barriers and enhancing understanding of RNA regulatory networks are essential for future success.

## 1. Introduction

RNA therapeutics represent one of the most dynamic and promising frontiers in modern biomedical research, enabling precise gene modulation previously unattainable with traditional small molecules or protein-based drugs. The prospects of such approaches are evident due to changes in the cell population at the genetic level, which necessitates the development of agents acting at the same hierarchical level. From this point of view, oncological pathologies, virological diseases, and some bacterial and parasitic infections are similar in their hierarchical level, i.e., they introduce changes in the normal functioning of the cell population at the level of nucleic acids, intracellular signalling cascades, and protein–protein interactions. Disruptions in the normal transformation of information within the cell and the transport of various substances are the cornerstone for this set of pathological processes. Therefore, there is a need to find means by which these changes can be compensated for or the native mechanisms of life activity can be restored [1]. Also, the hijacking and manipulation of host cellular pathways, such as intracellular signalling cascades, gene expression machinery, and metabolic networks, is crucial for both viral replication and cancer cell proliferation. RNA therapeutics offer a precise way to interfere with these processes, whether by targeting viral RNA/proteins or dysregulated host genes or RNAs involved in both disease states. Examining this dual application allows for the identification of conserved therapeutic vulnerabilities.

Using the cell’s internal mechanisms—in particular, the ability of RNA interference (RNAi) to suppress gene expression and the fine-tuning provided by endogenous microRNAs (miRNAs)—it is possible to target pathological genes or transcripts relatively precisely or to restore balance in intracellular pro- and anti-apoptotic signalling cascades that suppress tumour growth [2,3].

At the heart of almost every clinical problem discussed in this review is the delivery vector. Although LNP-based platforms dominate in systemic and vaccine applications [4,5], innovative, albeit more risk-associated, approaches, such as bacterial minicells (TargomiR) and various polymer conjugates, have been tested in oncology. An effective delivery system must protect the RNA molecule from the action of specific nucleases in the bloodstream, facilitate cellular uptake, ensure escape from endosomes, and provide release of the nucleic acid at the desired nuclear or cytoplasmic location. One of the most critical aspects of RNA therapy involves delivery vehicles. For a more detailed examination of this subject, the reader is invited to consult our review on the topic so as to avoid unduly increasing the scope of the present review [6].

In addition to the challenges of physicochemical delivery, there is also the issue of regulating miRNA activity, which can pose a threat to the viability of both the cell and the organism as a whole. miRNAs exert pleiotropic effects on the expression of entire clusters of genes, which creates unpredictable long-term risks in their application. For example, it is known that in the treatment of hepatitis C, inhibition of miR-122 is associated with the risk of developing hepatocellular carcinoma (HCC). Furthermore, it is known that the use of MRX34 is associated with pronounced toxicity.

This review summarises the information and knowledge accumulated recently on clinical studies of various RNA-based drugs for the treatment of viral infections and cancer. The considered RNA-based therapeutic classes include drugs based on small interfering RNA (siRNA) and miRNA. RNA therapeutics function through distinct molecular mechanisms: siRNAs induce sequence-specific cleavage of target mRNA via the RISC complex; miRNA mimics restore lost tumour-suppressive functions; miRNA inhibitors sequester oncogenic miRNAs; and antisense oligonucleotides (ASOs) modulate gene expression through RNase H-mediated degradation or steric blocking.

The rationale for combining viral infections and cancer in this review is associated with their shared hierarchical level of pathogenesis, involving disruptions in intracellular signalling cascades and genetic regulation. Breast cancer is considered as a case study due to its high global disease burden and the diversity of identified RNA targets (e.g., Bcl-2, KRAS) that exemplify the complexity of miRNA-mRNA regulatory networks.

In addition to the topics covered by existing scientific literature that focus either on viral vaccines or on specific oncological targets, this review explores shared challenges and distinct applications of RNA therapeutics in viral infections and cancer. We bridge the gap between these fields by analysing shared molecular challenges, such as the complexity of miRNA-mRNA regulatory networks and the efficiency of diverse delivery systems (LNPs, GalNAc, and bacterial minicells). By framing breast cancer as a comprehensive case study, we illustrate the predictive challenges of systemic RNA interventions that are applicable across various solid tumours.

Despite advancements in the use of RNA-based therapeutic methods for oncology and viral diseases, uncertainties persist regarding their side effects—which may be short-term, medium-term, or long-term in nature. The former category includes, for instance, a typical acute immune response (manifesting as local irritation, fever, or general malaise); the latter encompasses delayed adverse events, such as the development of delayed-type allergic reactions or specific organ damage. Long-term side effects, meanwhile, remain insufficiently studied but pose the greatest risks from a population-level perspective. Indeed, long-term side effects have not yet been fully investigated due to the limited duration of follow-up periods. By critically evaluating the fate of specific siRNA, ASO, and miRNA-based agents, we aim to identify recurring patterns associated with systemic miRNA modulation and the varying efficacy of different delivery technologies. RNA-based therapeutic methods—whether applied in oncology or for infectious diseases—utilise similar operating principles, nucleic acid modification strategies, and delivery vehicles. Consequently, their associated side effects and limitations can be examined in tandem. Moreover, it is worth noting that certain agents (e.g., saRNA MTL-CEBPA and ASO-SP-101) are employed in both oncological and antiviral contexts, underscoring the hypothetical potential for similar drugs to address both types of diseases. Consequently, this highlights the need for a review that adopts this specific conceptual approach, focusing on the molecular and biological mechanisms that govern the action and development of these therapeutic agents.

## 2. RNA Therapeutics

### 2.1. RNA Therapeutics for Treatment and Prophylactics of Viral Infections

**Figure 1:**
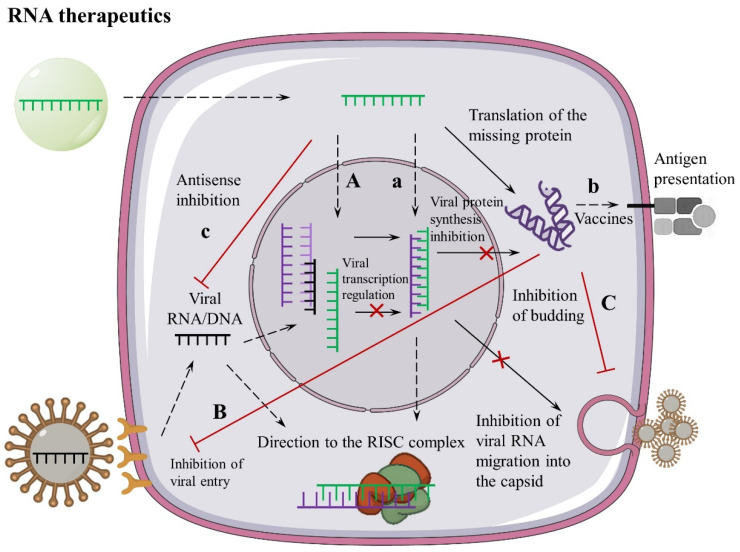
Potential and established molecular mechanisms and targets in the treatment and prevention of viral infections, with examples of RNA-derived drugs. Lowercase letters denote targets and mechanisms for which RNA-based drugs have already been developed and are undergoing clinical testing, while uppercase letters denote potential ones. a—regulatory potential of pathological RNA inhibition; drugs (e.g., miravirsen, temavirsen) antisense; b—antigen presentation through MHC complex or expression intercellular space (vaccines, e.g., BG505 MD39.3, BNT162b2, mRNA-1273); c—antisense inhibition of viral nucleic acid (e.g., JNJ-3989, VIR-2218); and A—gene editing and regulation of gene expression; B—inhibiting viral entry or release of viral nucleic acid from capsid; C—targeting the viral capsid budding process as future targets and mechanisms for drug development.

Despite developments in pharmaceutical technology, viral infections remain a major public health problem worldwide [7,8]. The most prevalent viral infectious agents in the 21st century are hepatitis B and C viruses, HIV, and SARS-CoV-2 (in 2020 and beyond). A summary of cellular mechanisms is shown in Figure 1.

Sandra et al. (2024) report over 350 million people living with HBV worldwide and more than 1 million annual deaths [9]. Despite the availability of effective vaccines against HBV, which have reduced the incidence of the disease in Europe, the United States and other developed countries, about 45% of the world’s population lives in regions highly endemic for hepatitis B, where the lifetime risk of infection with this infectious agent exceeds 60%. To avoid rapid disease progression leading to cirrhosis or liver carcinoma, chronically infected HBV patients must take nucleotide analogues [10]. In mouse models expressing the major HBV surface protein HBsAg (responsible for liver pathology), siRNA delivery targeting the corresponding transcript significantly reduced HBsAg levels and aided normal liver function maintenance [11,12,13,14,15,16,17]. Thus, HBV is an example of a DNA virus that can be controlled by targeting the corresponding RNAs. Researchers often used similar siRNA sequences targeting HBsAg mRNA production across the studies: a total of 11 RNA variants were tested, and cholesterol liposomes, lipid nanoparticles, or covalently attached GalNAc were used for delivery. Despite reported successes, clinical trial data or market availability for these agents are largely absent with rare exceptions. For example, Phase II clinical trials for ARC-520 were prematurely terminated due to significant adverse effects, specifically cardiorespiratory events observed in non-human primates during preclinical safety studies. These events were attributed to the delivery vehicle (ARC-EX1), a lipid nanoparticle formulation, which was shown to induce a complement-mediated immune response leading to mast cell degranulation and histamine release, rather than a toxicity of the RNA therapeutic itself [18,19]. Subsequently, it was demonstrated that the delivery agent ARC-EX1 induces histamine release through mast cell degranulation [20].

The situation is similar for the hepatitis C virus (HCV), except that there is no prophylactic vaccine against it. El-Tahan et al. report HCV leads to more than 350 thousand deaths each year [21]. About 75–85% of infected people have persistent viral infections, and only about 20% are completely cured. About 5–20% of chronically infected HCV patients develop cirrhosis, of whom ~25% develop hepatocarcinoma [22]. Previously the common therapy for HCV infection was combinatorial therapy with PEGylated interferon α in combination with ribavirin (PEGylated IFN-alpha plus ribavirin) [23]. However, up to 30% of patients do not reach recovery in this way [24]. In the study by Brjalin V et al. [25], the authors reported that the rate of the sustained virologic response (SVR) is associated with the viral genotype. In particular, the overall rate of SVR for patients from Estonia reached 60.3%; SVR was achieved in 46.1% of patients with HCV genotype 1b compared to 84.4% of patients with HCV genotype 3a. Therefore, the drugs that allow achieving SVR in patients infected with HCV genotype 1b are most in demand. The common therapy for HCV infection has evolved significantly; highly effective direct-acting antivirals (DAAs) have now become the standard of care, achieving high cure rates.

The inhibitors of viral enzymes may have a high probability of side effects and toxicity. Therefore, the methods of targeting the RNA of the virus and the host using silencing with complementary RNA and RNA interference are under development [26]. Small RNAs miR-155 and miR-122 appear in the literature as host cell targets, whose suppression may have a favourable effect on the HCV infection [27,28]. Suppression of miR-155 by U6-miR-155-TuD based on the pRNA-U6.1/Neo-small interfering Firefly luciferase (siFluc) vector significantly reduces the risk of hepatocarcinoma [29], and the loss of expression or inhibition of oncosuppressor miR-122 reduces the efficiency of HCV replication in liver cells. Meanwhile, suppression of miR-155 and miR-122 small RNA function had no adverse effects on the general condition of model animals. Delivery of short hairpin RNAs (shRNAs) can be used for disrupting the expression of virus and host genes important for maintenance of HCV infection [30,31]. Thus, it was in the study by Chen et al. where the authors demonstrated the suppression of p38 mitogen-activated protein kinase (MAPK) expression or phosphorylation causes impaired replication of HCV as well as several other DNA- and RNA-containing viruses—fever with thrombocytopenia syndrome virus (SFTSV), herpes simplex virus type 1 (HSV-1) and severe acute respiratory syndrome coronavirus 2 (SARS-CoV-2) [32]. Braga et al., in in vitro experiments, showed that HCV replication is effectively inhibited by the delivery of shRNA specific to the heat shock protein Hsp90 into cells [33]. The antiviral effect is particularly pronounced if this effect is combined with the delivery of shRNA specific to the highly conserved 5′UTR region of the viral genome. In the study by Youssef et al., the authors showed that transfection with shRNA specific to the 5′UTR of domain IIIC within the internal ribosome entry site, in combination with treatment with pegylated IFN-alpha plus ribavirin 24 h after treatment, resulted in a significant reduction in HCV genomic RNA load and complete disappearance of the key non-structural protein NS5A HCV [34]. Evidence suggests that HCV can rapidly and effectively evade the effects of siRNA (hypothetically due to several mechanisms and features: high genetic variability; RNA secondary and tertiary structure—much of the HCV genome is folded into complex secondary structures like hairpins, pseudoknots, and internal ribosomal entry sites; intracellular compartmentalisation—HCV remodels host cell membranes to create compartments that serve as replication sites where viral RNA is synthesised and housed; the production of viral RNA in an infected cell is massive and rapid in the way that the volume of viral RNA significantly exceeds the capacity and processive abilities of RISC complexes within the cell); this should be considered when developing therapeutic agents using this approach [35].

Based on available in vitro trial data, Santaris Pharma developed miravirsen (anti-miR-122) for the treatment of chronic hepatitis C. This drug is intended primarily for patients unresponsive to standard combination therapy with PEG-interferon α and ribavirin. Miravirsen is an LNA/DNA phosphorothioate antisense oligonucleotide, 15 nt in length, complementary to miR-122, which forms a stable heteroduplex with mature miR-122, thereby inhibiting its function. As discussed in the previous chapter, miravirsen was investigated as a therapy for HCV [36,37].

Miravirsen was approved by the FDA to enter clinical trials in 2010 and underwent Phase I trials in 2012–2014 but has not been authorised for use in any country in the world to date.

In 2017, the developer began Phase II clinical trials [36]. No information is available about further testing and obtaining authorisation for use. In in vitro cell culture trials, miravirsen was equally effective in inhibiting HCV replication of HCV genotypes 1–6. It is active against strains that have developed resistance to DAAs (direct-acting antivirals). In a telaprevir (DAA, telaprevir inhibits the hepatitis C viral enzyme NS3/4A serine protease) resistance test, isolates with resistance to telaprevir were detected in patients 25 days after starting this DAA. With miravirsen (80 μM), no isolates with mutations were detected after 155 days. A Phase IIa clinical trial of miravirsen, conducted at seven international centres, evaluated the safety and efficacy of the drug on a group of 36 patients with chronic HCV genotype 1 infection. Trial organisers observed no side effects requiring dose limitation of the drug and no mutations in miR-122 binding sites in the HCV genome [38].

In a Phase IIa clinical trial (SPC6649-203) of miravirsen, dose-dependent effects on viral RNA content in plasma and liver were confirmed. As a side effect, a decrease in cholesterol levels was detected in patients in the experimental groups. No significant changes were found in the miR-122 binding region (i.e., except for baseline changes—pre-existing polymorphisms present before treatment) [39].

Given that HCV infection significantly increases the risk of hepatocellular carcinoma, a significant issue arising in trials of miravirsen is the assessment of its effect on the course of this cancer. MiR-122 has been reported to be involved in the development of HCV infection [40], HCC [41], and treatment resistance of HCC [42]. However, from the above data, it appears that suppression of miR-122 expression, while providing reduced viral load in HCV patients, significantly increases the risk of hepatocellular carcinoma. A number of genes, such as Snail 1 and Snail 2, responsible for epithelial–mesenchymal transition, the WNT1 gene from the WNT signalling pathway, and CREB1 and BCL9, whose expression is regulated by miR-122, are known. There is evidence that the long non-coding RNA ANRIL and the RNA-binding protein AUF1 suppress miR-122 expression, promoting HCC progression; the regulation of miR-122 expression remains poorly understood. Most studies on the interaction between lncRNA and miR in HCC consider lncRNAs as miR sponges that share the same binding sequences of certain miRs with mRNAs and compete with them for miR uptake. A CpG island in the promoter region of miR-122, which is involved in the regulation of miR-122 expression, was identified [43]. Thus, DNA methylation, along with regulation of miRs expression, is a key epigenetic factor affecting HCC progression. Authors presented the results of methylome profiling of samples from 304 HCC patients who underwent surgical resection and confirmed the presence of 36 DNA methylation biomarkers that accurately predict poor survival, indicating that DNA promoter methylation may predict prognosis in HCC [44]. These data provide new insights into the mechanisms of mutual regulation of two key non-coding RNAs in HCC: HOTAIR and miR-122, and suggest the negative regulatory axis of HOTAIR/miR-122/Cyclin G1 as a promising molecular target for HCC treatment. The work by Cheng et al. found that lncRNA HOTAIR, which activates the repressive polycomb 2 complex with its histone H3 methylation activity, is overexpressed and reduces miR-122 expression in HCC tissues [43]. As a well-known oncogene in most tumours, both in vitro and in vivo experiments showed that HOTAIR knockdown inhibits HCC cell proliferation, induces cell cycle arrest and suppresses tumour progression through negative regulation of miR-122. Taken together, the present data suggest that due to the lack of knowledge about the regulation of miR expression in various tissues and organs, the use of drugs that affect their behaviour may cause unpredictable consequences for the patient. The authors have compared the results of studies on siRNA- and miRNA-based drugs and concluded that the primary limitation of miRNA-based therapy lies in the fact that it typically targets a large number of targets (tens or hundreds of genes), which is practically impossible to control in practice [45].

Human immunodeficiency virus has long remained in the centre of attention of biomedical specialists around the world. In 1999 the article suggested the possibility of creating an siRNA-based drug for HIV therapy [46]. Subsequently, the results of trials of siRNA suppressing the expression of the cellular gene TSG101, which is necessary for the detachment of HIV particles from the infected cell, were described [47] as well as the induction of viral transcript degradation [48,49]. However, from the first steps in the development of siRNA-based HIV therapeutics, researchers encountered a rapid decline in its efficacy due to the mutability of the viral genome [50]. Therefore, as in the case of HCV, it has been proposed to select host cell transcripts essential for the maintenance of the virus life cycle, combining them with virus-specific siRNAs, as well as to use multiple virus-specific siRNAs simultaneously as a target for RNA interference. Authors report the creation of a computer programme that allows the calculation of siRNA efficiency parameters, taking into account the available information on its interaction with the PAZ and PIWI domains of the Argonaute nuclease and the RISC complex [51]. As a result, 50 siRNA variants were selected and tested, which showed high efficacy in suppressing HIV replication in peripheral blood monocytes of patients with HIV. The authors suggested that the simultaneous use of multiple siRNAs specific to different parts of the virus genome may help prevent the emergence of mutations that allow the virus to avoid the effect of the drug. Authors report that mRNA vaccines offer a promising approach for HIV vaccine development, for example, by delivering membrane-bound Env trimers (BG505 MD39.3) to enhance B-cell response, which can elicit stronger neutralising antibody responses and reduce off-target base-directed responses compared to soluble Env trimers and show promising results in Phase I clinical trials [52]. Negative side effects included chronic urticarial reactions [53,54].

The pandemic caused by the SARS-CoV-2 virus in 2019–2022 has become the most serious challenge to the global health system ever, affecting all countries without exception [55]. Advances in RNA delivery have enabled the rapid development of RNA vaccines to prevent this infection: Spikevax (Moderna, Cambridge, MA, USA) and BNT162b2/Comirnaty (Pfizer, New York, NY, USA/BioNTech, Mainz, Germany/Fosun Pharma, Shanghai, China). Moderna’s COVID-19 vaccine contains a 4101 nt long mRNA encoding a full-length S-protein with mutations (K986P and V987P) designed to stabilise the conformational state of the product, surrounded by the 5′-UTR and 3′-UTR of the human alpha-globin (HBA1) gene and poly-A [56]. The peculiarity of mRNA is the use of 1m-pseudouridine instead of uracil, which decreased the immunogenicity of the molecule and its recognition efficiency by TLR and increased translation efficiency [57]. A method for obtaining mRNA modified in this way using in vitro transcription is described in [58]. The mRNA is packaged in liposomes containing the following lipids: SM-102, PEG2000-dimyristoylglycerol (DMG), cholesterol, 1,2-distearoyl-sn-glycero-3-phosphocholine (DSPC), tromethamine, and tromethamine hydrochloride. This composition of lipid nanoparticles was first proposed in [59]. The BNT162b2 vaccine comprises a 4284 nt long mRNA containing the following elements: cap (m7G+m3′-5′-ppp-5′-Am)- 2 nt, 5′-UTR (51 nt), signal peptide sequence (58 nt), S-protein (3777 nt), 3′-UTR (295 nt), and poly-A (110 nt).

As with the Spikevax vaccine, the mRNA in the BNT162b2 vaccine composition contains 1m-pseudouridine instead of uracil. In addition, the vaccine contains lipids (4-hydroxybutyl) azandiyl) bis (hexane-6,1-diyl) bis (2-hexyl decanoate), 2[polyethylene glycol-2000]-N, N-ditetradecylacetamide, 1,2-distearoyl-sn-glycero-3-phosphocholine, and cholesterol [60]. It should be noted that all the main approaches used in the development of vaccines against SARS-CoV-2 were previously worked out on the vaccine model for the prevention of Zika virus infection [61]. The developers of the Spikevax and BNT162b2 RNA vaccines against SARS-CoV-2 state that immunisation provides 95% protection against virus infection. However, using mRNA without replacing uracil with 1m-pseudouridine resulted in only 48% protection [62].

Furthermore, the development of RNA-based therapies for COVID-19 heavily emphasised aerosol delivery methods, targeting the respiratory tract epithelium directly. This approach is based on the understanding that SARS-CoV-2 primarily replicates within this region of the human body [63]. Given the propensity of viruses to rapidly evade nucleotide-specific drugs through mutagenesis, predominantly host transcripts critical for virus replication but conditionally important for maintaining normal host function were selected as targets [64]. In addition, siRNA delivery has been used to reduce the severity of COVID-19 symptoms [65]. For example, in [66] the authors used a specific siRNA that targets TNF-α, combined with a cell-penetrating peptide SAR_6_EW, which improved intracellular delivery, resulting in gene silencing in in vivo and in vitro experiments. Given that TNF-α hyperproduction in SARS-CoV-2-infected cells is a key driver of lung tissue damage, such an agent could effectively prevent mortality in severe COVID-19 patients during the acute phase of the disease.

### 2.2. RNA Therapeutics for Treatment of Tumours

**Figure 2:**
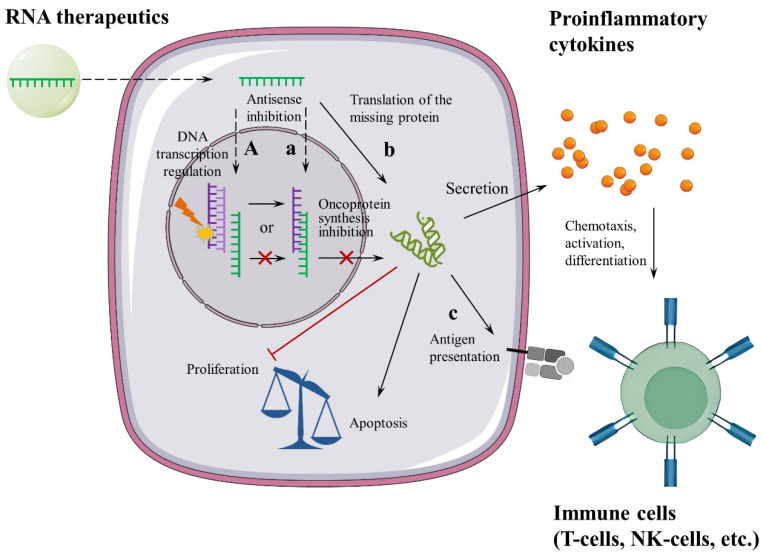
Potential and established molecular mechanisms and targets in the treatment and prevention of oncology, with examples of RNA-derived drugs. Lowercase letters denote targets and mechanisms for which RNA-based drugs have already been developed and are undergoing clinical testing, while uppercase letters denote potential ones: a—antisense inhibition of tumour RNA (e.g., TargomiR, cobomarsen); b—translation of the missing protein (e.g., MTL-CEBPA, RAG-01); c—antigen presentation (e.g., BNT-122, mRNA-4157); and A—gene editing and regulation of gene expression as future targets and mechanisms for drug development.

A summary of biological activity mechanisms of RNA-derived drugs in oncology is shown in Figure 2.

#### 2.2.1. TargomiR (miR-16 Mimic-Based Therapy) in Mesothelioma and Non-Small Cell Lung Cancer

TargomiR was developed by the Australian company EnGeneIC Limited as a treatment for malignant pleural mesothelioma and non-small cell lung tumours [67]. TargomiR is a system consisting of three components: (i) MesomiR-1, a miR-16 mimic (double-stranded synthetic RNA, 23 bp); (ii) bacterial minicells (EnGeneIC Dream Vector (EDV^TM^), EnGeneIC Dream Vehicles) as the delivery vehicle; and (iii) bispecific antibodies on the minicell surface mediating tumour-targeted delivery (e.g., via EGFR). The natural function of miR-16 is to negatively control epidermal growth factor receptor (EGFR) expression. In lung tumours as well as some others such as bladder cancer [68], miR-15 and miR-16 deficiency is observed, contributing to their malignancy. The active ingredient TargomiR is a double-stranded synthetic RNA molecule MesomiR-1, of 23 base pairs in length. A unique feature of TargomiR is that EDV^TM^ are non-viable bacterial minicells coated with bifunctional tumour-specific antibodies [69].

Previously, researchers described *E. coli* mutants of the MinC-MinD-MinE gene cluster producing a large number of non-viable minicells with a diameter of ~400 nm [70,71]. Subsequently, they described a reliable method of preparative purification of chromosome-free and non-viable minicells of *E. coli* and *Salmonella typhimurium* from viable bacteria by incubation of the grown culture in medium with 5% NaCl for 4 h followed by tangential nanofiltration through a filter with a pore diameter of 0.45 nm [72]. The same work described a method of loading minicells with chemopreparations and short RNAs by incubation with solutions of active substances with a concentration of 250–400 µg/mL.

Due to the unique properties of the porin system embedded in the outer membrane of minicells, the transport of hydrophobic molecules is effectively unidirectional: drugs that penetrate minicells are completely retained within them during 72 h of incubation at room temperature or after 4 months of frozen storage. Purified and chemo-loaded minicells were coated with bifunctional antibodies targeting the O-polysaccharide component of LPS and human EGFR, HER2, CD33, and CD3 receptors, which are specific to many tumour types. This ensures efficient targeting of minicells to these tumour types. The diameter of the minicells (~400 nm) also facilitates efficient penetration into tumours in vivo via the enhanced permeability and retention (EPR) effect.

Authors described a method of packaging synthetic siRNA into minicells, which allows reduction in the concentration of transcripts significant for tumour survival, in particular, EGFR [73]. The design of a minicell-based antitumour agent closely resembling TargomiR and presented positive results of its trials on malignant pleural mesothelioma xenografts in SCID (severe combined immunodeficiency) mice were also described [74].

In addition, experiments on the MPM (malignant pleural mesothelioma) cell line showed restoration of the miR-16 expression level in it, which was initially reduced about 10-fold compared to the MeT-5A line of normal lung mesothelial cells.

Phase I clinical trials of TargomiR were conducted in Australia between 2014 and 2017 on a cohort of 27 patients. The results were deemed unsatisfactory in terms of observed treatment efficacy, and the TargomiR trial was discontinued in 2017.

#### 2.2.2. Cobomarsen (Anti-miR-155) in T-Cell Leukaemia/Lymphoma/Mycosis Fungoides

Cobomarsen is an LNA-modified antisense oligonucleotide miR-155 inhibitor (antimiR) drug for the treatment of T-cell leukaemia, lymphoma and fungal shingles. Its originator is miRagen Therapeutics, and its developer is Viridian Therapeutics. Cobomarsen was tested in patients with different types of lymphomas and leukaemias, including mycosis fungoides (MF), chronic lymphocytic leukaemia (CLL), activated B-cell lymphoma, and adult T-cell leukaemia/lymphoma (ATLL).

Cobomarsen is an LNA-based drug for subcutaneous or intravenous injection. It should be noted that the developers of cobomarsen do not inform the scientific community about the sequence of the oligonucleotide they use, as well as the composition of the components of the LNA-modified antisense oligonucleotide packaging system.

The cutaneous T-cell lymphoma Phase I clinical trial of cobomarsen was completed in 2018 [75]. At the same time, Phase I for ATLL in the USA showed promising results [76]. However, Phase II was terminated in 2020. Phase I for ATLL and Phase I for CLL have been discontinued [77]. It was proposed that these terminations were driven by strategic business decisions by the sponsor to reprioritise their R&D pipeline, rather than the emergence of unacceptable toxicity profiles or definitive lack of efficacy.

There are published results from the developer trials of cobomarsen. Overexpression of miR-155 in blood leukocytes is statistically associated with poor prognosis in lymphoma and leukaemia, as well as with the progression of mycosis fungoides, the most common form of cutaneous T-cell lymphoma (CTCL). An in vitro study of skin biopsy specimens from patients with mycosis fungoides demonstrated that cobomarsen-mediated suppression of miR-155 expression reduced the expression of multiple gene pathways associated with the survival of abnormally behaving cells, reduced survival signalling, decreased cell proliferation, and activated apoptosis [78]. A set of genes significantly regulated by cobomarsen was identified. Using biopsy specimens from MF patients and CTCL cells infected with human T-lymphotropic virus type 1 (HTLV-1), researchers showed that miR-155 expression simultaneously regulates multiple parallel malignant cell survival pathways (including JAK/STAT, MAPK/ERK, and PI3K/AKT) previously associated with MF pathogenesis and that these survival pathways are inhibited by cobomarsen in vitro. In a Phase I clinical trial of cobomarsen in patients with CTCL, methods were used to determine the dynamics of identified biomarkers to assess the pharmacodynamic response to therapy.

The authors reported that miR-155 is an oncogenic microRNA highly expressed in B-cell malignancies, especially in the B-cell subtype of germinal centreless diffuse large B-cell lymphoma or activated B-cell subtype of diffuse large B-cell lymphoma (ABC-DLBCL), where it is considered a potential diagnostic and prognostic biomarker [79]. Thus, inhibition of miR-155 represents an important therapeutic strategy in B-cell lymphomas. The authors of this work tested the efficacy and pharmacodynamic activity of the miR-155 oligonucleotide inhibitor, cobomarsen, in ABC-DLBCL cell lines and in relevant xenograft mouse models. In addition, they evaluated the therapeutic efficacy and safety of cobomarsen in a patient diagnosed with aggressive ABC-DLBCL. Intravenous injections of cobomarsen into NSG (NOD scid gamma) mice bearing ABC-DLBCL xenografts were also performed to study tumour growth and pharmacodynamics of the compound over time. Cobomarsen reduced cell proliferation and induced apoptosis in ABC-DLBCL cell lines. Intravenous administration of cobomarsen to a mouse model of ABC-DLBCL with NSG xenograft reduced tumour volume, triggered apoptosis, and decreased direct expression of miR-155 target genes. Finally, the compound reduced and stabilised tumour growth without any toxic effects on the patient. Results confirm the efficacy of cobomarsen in ABC-DLBCL and other types of lymphoma with increased miR-155 expression [79].

Multi-year trials (2020–2024) of cobomarsen have not substantiated its practical efficacy, likely because particles containing the active substance fail to efficiently penetrate human tumour cells. This assumption is based on the fact that the adequacy of miR-155 selection as a target and the ability of the oligonucleotide to inhibit its pathologic activity have been convincingly proven in experiments on models, while the efficacy of the active substance delivery in humans has not been evaluated in clinical trials for obvious reasons. There are reasons to think that it may differ in the case of humans and mice since there is evidence that the effect of EPR in the case of mouse tumours is significantly more pronounced than in humans [80,81].

#### 2.2.3. MRX34 Against Liver Cancer, Lymphoma and Melanoma

The drug MRX34, developed by Mirna Therapeutics Inc., is intended for the therapy of melanoma, as well as liver cancer and lymphoma. In [82], it was reported that MRX34 is a 23 nt long double-chain synthetic miR-34a mimetic (mimic miR-34a) encapsulated in an LNP with a diameter of ~110 nm. LNPs contain amphoteric lipids, which acquire a positive charge when liposomes enter the acidic environment of cellular lysosomes. At the same time, at neutral pH in the blood and intercellular medium, liposomes have anionic properties, which prevent the aggregation of particles and their adhesion to endothelial cell membranes [83]. MRX34 exhibits prolonged circulation in the blood, which the developers claim facilitates efficient delivery of the miR-34a mimetic to tumours, liver, bone marrow, spleen, lungs, and other tissues following intravenous administration in mice and monkeys [84]. MicroRNA-34a (miR-34a) is a naturally occurring tumour suppressor that suppresses the expression of over 30 different oncogenes, as well as genes that enable tumours to evade recognition by the immune system. In many malignancies, miR-34a expression is reduced or absent.

Phase I clinical trials of MRX34 were designed to evaluate the maximum tolerated dose and safety of the drug, as well as to investigate its pharmacokinetics and clinical efficacy in patients with advanced solid tumours. The trial enrolled 48 adult patients with solid tumours resistant to standard treatment, including 14 patients with HCC. MRX34 was administered intravenously twice weekly for three weeks in cycles of 4 weeks. The mean age of the patients was 60 years, and the mean duration of prior treatment was 4 years (1 to 12 years). The most common side effects were fever (64%), fatigue (57%), back pain (57%), nausea (49%), diarrhoea (40%), anorexia (36%), and vomiting (34%). Abnormalities detected by laboratory tests included lymphopenia 23%, neutropenia 13%, thrombocytopenia 17%, aspartate aminotransferase elevation 19%, hyperglycaemia 13 and hyponatraemia 19%. Dexamethasone premedication was required to prevent infusion-related adverse effects. The maximum tolerated dose for patients without HCC was 110 mg/m^2^, and hypoxia and enteritis were reported in two patients at doses greater than 124 mg. The half-life of the active ingredient MRX34 was >24 h, and Cmax (peak serum concentration of a drug) and AUC (area under the curve) contrasting blood concentration increased with increasing dose. One patient with HCC achieved a durable confirmed response at 48 weeks after trial initiation.

The developers claimed that MRX34 therapy after premedication of patients with dexamethasone provided them with an acceptable level of toxicological safety with oncostatic effects against advanced solid tumours. The recommended daily dose for non-HCC patients was 110 mg/m^2^ and for HCC patients was 93 mg/m^2^. Additional MRX34 administration regimens have been studied to improve tolerability. However, the results of the clinical trials in terms of the efficacy of the therapy were considered unsatisfactory, so the MRX34 trial was discontinued in 2016.

#### 2.2.4. A Tumour Therapy Drug Based on miR-221 Inhibitor

The authors reported the results of trials of a drug candidate based on a 13 nt long LNA-oligonucleotide, which is a miR-221 (LNA-i-miR-221—a 13-mer LNA antisense oligonucleotide with a fully phosphorothioate-modified backbone, without additional delivery system) complement based on a phosphorothioate skeleton of the main chain [85]. An animal model experiment demonstrated its ability to inhibit miR-221 production and antitumour activity against human xenografts in immunodeficient mice. The drug was claimed by the developer to have acceptable adverse toxicity against rats and monkeys. Interspecies scaling allowed the selection of an initial dose of LNA-i-miR-221, which was used in the subsequent Phase I clinical trial.

In this Phase II human trial, patients were shown to tolerate well four consecutive daily injections of LNA-i-miR-221 at a dosage of 5 mg/kg. No clinically significant changes in vital signs, physical examination findings or electrocardiogram were observed. No major safety concerns were noted in this trial, and the minimum toxic dose was not reached.

Analysis of LNA-i-miR-221 concentration kinetics in the plasma of patients showed that it exhibits a nonlinear decrease in the range of doses studied. Similar observations were obtained during preclinical safety studies conducted on rats [86,87].

The terminal half-life of LNA-i-miR-221, based on plasma concentration, ranged from 1.1 to 4.9 h and increased with increasing dose. However, LNA-i-miR-221 was detectable in urine within 2 days of the last dose in all patients in all groups. This suggests that the true half-life of LNA-i-miR-221 is significantly longer than that determined from plasma concentrations.

No significant changes in clearance rates were observed after repeated administrations. The excretion of LNA-i-miR-221 in urine at later time points suggests retention of a substantial fraction of the nucleotide in tissues. This observation aligns with previously published data indicating that modified nucleotides with phosphorothioate bonds can bind strongly to components of blood serum and various intracellular proteins [88,89].

Importantly, systemic exposure to LNA-i-miR-221 resulted in decreased expression levels of miR-221 and increased expression of CDKN1B mRNA and protein, as well as PTEN. Thus, even an unpackaged nucleotide can influence the expression level of the target gene in blood cells. These data demonstrate that the investigational drug exhibits biological activity against miR-221 targets and that this activity correlates with an increase in patients’ pharmacokinetics receiving higher doses [90].

Of the 16 patients receiving LNA-i-miR-221 during this study (refractory advanced cancer) in whom clinical response could be assessed, the majority had stable disease (8 patients), as determined by computer tomography imaging of tumours over time.

LNA-i-miR-221 is an investigational drug that has shown potential in treating various cancers, including hepatocellular carcinoma, breast cancer, glioma, prostate cancer, pancreatic cancer and multiple melanoma.

#### 2.2.5. RNA-Derived Therapeutics for Breast Cancer Therapy

Breast cancer is the most common type of tumour in women worldwide. GLOBOCAN reports that there were 2.3 million primary diagnosed cancers in 2020, representing 11.7% of all newly diagnosed cancers [91]. In 2020, breast cancer was responsible for 684,996 deaths. In 2020, the age-standardised incidence rate (ASIR) and age-standardised mortality rate (ASMR) of breast cancer ranked first and second among all cancers, respectively [92]. Therefore, the development of breast cancer therapeutics is of critical socioeconomic importance. Based on the expression profile of key markers, oestrogen receptor (ER), progesterone receptor (PR), human epidermal growth factor receptor 2 (HER2), and Ki67, breast tumours are divided into the following types: luminal A, luminal B, luminal/HER2, HER2 enriched, basal-like, and triple negative (TN) nonbasal [93]. Each type has specific clinical features and requires different treatment modalities. These types serve as suitable models for demonstrating the intricate complexity of miRNA-mRNA regulatory networks and illustrating how interconnected regulatory axes involving oncogenes like c-myc and specific miRNA clusters impact treatment resistance and disease progression. Consequently, while breast cancer is used here to exemplify broader challenges in RNA therapeutic development applicable to various solid tumours, the review maintains equivalent depth for other major malignancies where RNA-based agents have reached clinical stages, such as malignant pleural mesothelioma (TargomiR), lymphomas and leukaemias (cobomarsen and 5-FU-miR-15a), and liver cancer and melanoma (MRX34).

Transcripts of the oncogenes Bcl-2, Kirsten rat sarcoma (KRAS), and c-myc are considered promising targets for the development of breast cancer therapy [94,95,96,97]. Bcl-2 is directly involved in the regulation of various cellular processes necessary for tumour growth, including stimulating tumour cell proliferation, their penetration through interstitial barriers, metastasis, neoangiogenesis, and the development of resistance to chemoprevention and radiation therapy. Increased expression of Bcl-2 is observed in most tumour types, including breast carcinoma, and the degree of Bcl-2 overexpression correlates with a poor prognosis for survival. Results of testing a siRNA-based drug that induces Bcl-2 transcript degradation in a human breast carcinoma xenograft model of triple-negative breast carcinoma in SCID mice are presented [98]. Complex colloidal composition was created by simply mixing siRNA specific to Bcl-2, benzethonium chloride (BZT) and pluronic F-68. In vitro and in vivo studies showed that the complex formed stable nanostructures with a diameter of less than 10 nm with a hydrophilic surface that provided (1) reliable shielding of siRNA from nucleases in plasma, (2) efficient transfection into tumour cells, and (3) primary accumulation in tumours due to the EPR effect. High antitumour activity of the tested drug based on the synergy of siRNA specific to Bcl-2 with traditional chemotherapeutic agents for breast cancer treatment was demonstrated.

In the study by Liu, J. et al., 2022 [99] the authors reported that elevated levels of miR-4458 were observed in ER-positive breast tumour cells. However, in vitro testing revealed that administration of miR-4458 to MCF-7 human breast carcinoma MCF-7 cells observed a decrease in their viability, colony formation and migration ability. Collagen type XI alpha 1 (COL11A1) has the ability to interact with miR-4458 directly. High COL11A1 expression is positively correlated with a negative prognosis of the course of ER-positive breast tumour type, lower overall survival, and increased risk of recurrence and metastasis to tumour nodes. Suppression of COL11A1 expression inhibited the migration of MCF-7 cells in culture. The authors used gelatin nanospheres to deliver the nuclease-resistant RNA mimetic miR-4458 and siRNA that suppresses COL11A1 expression (si-COL11A1) into human mammary tumour xenografts cultured in immunodeficient mice. After administration of the drug, a decrease in tumour volume was observed, apoptosis of tumour cells was accelerated, and metastasis to the liver was significantly delayed. In addition, administration of the drug inhibited the activity of the DDR2/SRC signalling pathway in xenografts. The therapeutic potential of miR-4458 lies in a dual-targeting approach using nuclease-resistant mimics alongside siRNAs against COL11A1. This strategy aims to simultaneously deactivate the DDR2/SRC signalling axis, effectively ‘locking’ the tumour cells in a non-invasive state and preventing metastatic seeding in ER-positive breast cancer.

Several studies have reported the possibility of using miR-155 as a target for therapy of HER2-positive breast tumour variants using advanced delivery systems to transport therapeutic short RNAs into tumour cells [100]. There are identified 113 differentially expressed microRNAs (DEMs) specific to HER2-positive breast tumours and 923 of their target genes [101]. Among them, 110 genes whose expression affects HER2-related drug resistance of tumours were identified. The most significant among them were Bcl-2, Fos and CXCR4. In [102] it is reported that miR-23a-5p has a complementary interaction site with the 3′-UTR of the HER2 transcript and that this small RNA plays an essential role in the occurrence of breast tumours with the HR+/HER2-low phenotype.

For miR-23a-5p, the proposed clinical approach focuses on its role in modulating HER2-low phenotypes. As this miRNA targets the 3′-UTR of HER2, its targeted inhibition could theoretically upregulate HER2 expression levels, potentially expanding the eligibility of patients for HER2-targeted antibody-drug conjugates (ADCs) like trastuzumab deruxtecan. This highlights a “priming” strategy where RNA therapeutics are used to modify the tumour phenotype before primary treatment.

The authors have found a significant increase in miR-660-5p levels in MDA-MB-231 and MCF-7 breast tumour cell lines compared to MCF-10a cells [103]. In in vitro experiments, suppression of miR-660-5p function resulted in decreased proliferation rate, cell migration and invasiveness rates, and angiogenesis in HUVEC (human umbilical vein endothelial) cells. Bioinformatic analysis was used to identify 15 potential targets for miR-660-5p.

Authors have reported an important role of miR-205 as a negative regulator of MED1 gene expression responsible for the development of drug resistance to oestrogen analogues used for therapy of ER (oestrogen receptor)-positive breast cancer [104].

Authors have provided a review of the data on the analysis of the role of the long antisense transcript SOX2-OT as an oncogene that promotes the development of various types of tumours, including breast tumours [105]. More than 20 different miRs have been shown to be involved in the regulation of His SOX2-OT expression. This example shows the difficulty of predicting the action of each individual miR, which may have many uncharacterised targets along with known ones.

#### 2.2.6. Other Approaches for RNA-Based Therapy

The RNA therapeutic 5-FU-miR-15a (also known as CR1-02) is a modified miRNA mimic that integrates the cytotoxic pyrimidine 5-fluorouracil into the miR-15a sequence to treat acute myeloid leukaemia (AML) by suppressing oncogenic pathways such as BCL-2 and WEE-1 [106]. This drug is currently being evaluated in a Phase I, open-label, multi-centre dose-finding and expansion study to investigate its safety, tolerability, and preliminary efficacy in patients with relapsed or refractory AML [107]. Another candidate, 1B3 (or INT-1B3), is a lipid nanoparticle-formulated mimic of the tumour suppressor miR-193a-3p designed for the treatment of advanced solid tumours by inducing cell cycle arrest, apoptosis, and modulating the immune tumour microenvironment to turn “cold” tumours to “hot” ones. Preclinical and early clinical data for 1B3 demonstrate its ability to consistently suppress pro-tumourigenic phenotypes across various cancer cell lines and promote anti-tumour immunity by enhancing T-cell-mediated immune responses [108]. TTX-MC138 is a first-in-class therapeutic that uses an anti-miR-10b conjugated to iron oxide nanoparticles to inhibit miR-10b, a master regulator of metastatic cell viability [109]. Clinical experience from a Phase 0 microdose study (NCT05908773) has demonstrated that modified TTX-MC138 successfully accumulates in metastatic lesions in the bone, lung, and liver of patients with advanced solid tumours while maintaining a long circulation half-life and showing robust pharmacodynamic activity.

In summary, numerous target RNAs have been identified in the literature [110,111,112], the suppression of which demonstrates significant promise for tumour treatment in xenograft and in vitro models. However, complex network interactions among miRNAs, mRNAs, and long non-coding RNAs (including complementary mRNAs of oncogenes and tumour suppressors) make it exceedingly difficult to predict the physiological consequences of miRNA mimetics and inhibitors at the whole-organism level.

## 3. Discussion

Despite the numerous encouraging positive aspects associated with the use of RNA therapeutics, numerous challenges remain in their practical application, including potential negative effects. There are different theoretical possibilities of negative side effects (Table 1) of RNA therapeutics, which may be based on various molecular mechanisms such as interaction with other nucleic acids; potential negative side effects can arise from off-target binding, unintended immune responses to the RNA or delivery vehicle, interference with endogenous RNA functions, or systemic accumulation in non-target organs. Future research should address these potential challenges and test them in long-term clinical trials.

A review of RNA therapeutics for viral infections and cancer shows that this approach holds promise, but, like any therapeutic method, it has its drawbacks and challenges. The success of modified mRNA vaccines against SARS-CoV-2 demonstrates the feasibility of using liposomal nanoparticles and chemical modification to evade innate immunity. Similarly, the targeted use of GalNAc conjugates to suppress liver expression (relevant for hepatitis B virus) demonstrates the possibility of organ-specific targeting, even if the target organ has natural uptake mechanisms.

A key finding regarding the use of RNA-based methods (TargomiR, MRX34, cobomarsen) in oncology is the ineffectiveness of delivery to human solid tumours. Although preclinical models often demonstrate effective suppression or restoration of miRNA activity, translation to patient treatment currently faces insurmountable challenges. Furthermore, the discontinuation of MRX34 highlights the danger of toxicity; systemic administration of an agent designed to restore a general tumour suppressor (miR-34a) resulted in unacceptable systemic side effects, leading to the halt of trials, even despite some antitumour activity.

In this review, we have selected therapeutic candidates that represent different stages of the development (Table 2) to illustrate the transition from fundamental discovery to clinical application. The selection of specific RNA-based compounds was guided by three primary criteria: (1) agents that have reached Phase I/II clinical trials (e.g., TargomiR, cobomarsen, MRX34), serving as critical case studies for analysing delivery efficiency and systemic toxicity in humans; (2) candidates that target well-characterised oncogenic or viral pathways (e.g., miR-221, miR-15a), providing insight into novel chemical modification strategies demonstrating delivery vehicle efficacy; and (3) early-stage therapeutic targets identified in high-burden diseases such as breast cancer (e.g., miR-205, miR-23a, miR-4458), which exemplify the intricate complexity of miRNA-mRNA regulatory networks and are used specifically to illustrate the challenges of unpredictable off-target effects (imperfect sequence complementarity, unintended interactions with cellular machinery, immunogenicity of the RNA payload or delivery vehicle) and regulatory cascades. By covering this broad spectrum from early-stage potential to clinical-stage hurdles, we aim to provide a comprehensive hierarchical perspective on the current landscape and future challenges of RNA therapeutics.

Another equally important challenge is the biological complexity of regulatory networks. For example, miravirsen successfully suppresses HCV replication but also alters host liver homeostasis, potentially promoting the development of hepatocellular carcinoma through complex regulatory axes involving lncRNA and other oncogenes. This interdependence suggests that future therapeutic development must move beyond simple target identification and toward comprehensive systemic pharmacokinetic and pharmacodynamic modelling that considers the entire regulatory cascade affected by the intervention, not just the primary target.

While developing delivery approaches for RNA therapeutics in cancer and viral diseases, researchers must account for the distinct molecular mechanisms of these pathologies. Understanding these differences is crucial for evaluating the efficacy of RNA-based treatments and exploring their broader application potential. Given that viral infections can significantly alter biological pathways and cellular functions, analysing shared mechanisms may reveal the role of viral infections in oncogenesis [166,167,168]. Such insights could prove instrumental in designing novel therapeutic strategies for cancer, including RNA-based interventions.

Despite innovations in delivery platforms like lipid nanoparticles and GalNAc conjugates, their inherent challenges significantly complicate clinical translation. Due to immunogenicity, complex and costly manufacturing, and limited tissue tropism, these systems often fail to successfully cross significant biological barriers like the tumour stroma or blood–brain barrier. Moreover, therapeutic limitations and clinical trial failures are directly related to ongoing challenges in achieving effective endosomal escape and preventing off-target organ accumulation.

Finally, the inherent variability of viral genomes (e.g., HIV, influenza, herpes, adenoviruses, coronaviruses, etc.) requires a shift from single-target approaches to combined strategies using multiple siRNAs to minimise viral evasion. Directions involving exploitation of the body’s immunological mechanisms are particularly promising. Furthermore, the development of delivery systems that utilise active targeting mechanisms (beyond simple surface functionalisation) specific to tumour antigens is actively underway. The use of studies to correlate systemic exposure (LNA-i-miR-221 in blood cells) with therapeutic effect is also underway, and localised routes of administration (e.g., intratumoral or inhalational) that minimise systemic exposure are also being explored.

## 4. Conclusions

The successful deployment of mRNA vaccines against SARS-CoV-2 demonstrates RNA’s potential for rapid response and broad application. Conversely, persistent challenges in delivering RNA to solid tumours for cancer treatment contrast with vaccine applications. While promising preclinical data exist for various cancer targets, the frequent discontinuation of early-phase trials (e.g., MRX34, TargomiR) highlights that therapeutic potential is critically dependent on overcoming delivery barriers and managing systemic toxicity, issues less pronounced in vaccine applications.

The primary technical challenge remains the development of an effective and safe drug delivery system for infected or transformed cells (e.g., solid tumours). Expected clinical success depends heavily on the development of vectors capable of specifically penetrating target cells, organs, or tissues and ensuring intracellular release without significant systemic toxicity. At the same time, since infected cells include exogenous genetic information and cancer pathogenesis is associated with the disruption of cell transcription or translation, we should take this primary difference into account when estimating the whole possible set of the pharmacotherapeutic, toxic, and adverse effects of RNA therapeutic strategies for the treatment of cancer and viral infections. The complexity of biological systems, the potential for carcinogenic risks (potential for off-target effects that could contribute to oncogenesis and risks associated with prolonged inflammation), and unpredictable recombination (not a typical concern for mRNA and siRNA) of genetic constructs (especially in populations with complicated infectious backgrounds) raise legitimate questions and the need for more careful testing of candidate molecules.

## Figures and Tables

**Table 1:** Administration route, efficacy, and side effects for approved and investigational RNA-based drugs.

Drug Type	Names of Drug Examples	Disease	Administration Route	Efficacy	Side Effects	References
Self-amplifying RNA (saRNA)	ARCT-154, LNP-nCoVsaRNA, VLPCOV-01	COVID-19	Intramuscular (IM) injections	Neutralisation of SARS-CoV-2 15–48%	Headache, mild erythema and/or edoema, swelling, inflammation, fatigue, headache, myalgia, chills/rigours, arthralgia, nausea, fever	[113,114,115,116]
Circular RNA (circRNA)	RXRG001	Radiation-Induced Xerostomia-1	In the ducts of the parotid glands	Encodes human aquaporin-1	Cancer progression, genome instability, DNA damage, chronic inflammation, neurological disorders	[117,118,119]
RNA Editing Therapeutics	WVE-006, KRRO-110 (Korro Bio’s), and AX-1412 (ProQR Therapeutics’)	Alpha-1 Antitrypsin Deficiency, Cardiovascular diseases (AX-1412)	Intravenous, intrathecal, direct local, subcutaneous, inhalation, ex vivo modification	Correcting the SERPINA1 Z mutation, recruit endogenous ADAR, editing oligonucleotides to convert A-to-I for treating cardiovascular and liver diseases	Off-target editing, on-target toxicity, disrupt cellular processes, cancer progression, inflammation	[120,121,122,123,124,125]
siRNA Therapeutics	patisiran, givosiran, lumasiran, inclisiran, nedosiran, fitusiran, and vutrisiran	Hereditary transthyretin amyloidosis with polyneuropathy, acute hepatic porphyria, primary hyperoxaluria type 1, primary hypercholesterolaemia, haemophilia A or B	Intradermal injection, subcutaneous, intradermal Injection, intravenous, ophthalmic	Improved mNIS+7 neuropathy score vs. placebo at 18 months. Improved quality of life, mobility, and nutritional status; Reduction in urinary aminolaevulinic acid (ALA) and porphobilinogen (PBG). Reduction in annualised attack rate of acute hepatic porphyria (AHP) attacks vs. placebo; Reduction in urinary oxalate levels from baseline in adults; Reduction in LDL-C from baseline, on top of statin therapy. Effects sustained with biannual dosing; Reduction in oxalate production in children aged ≥ 9 years and adults with PH1; Reduction in bleeding rate. Maintained effective bleed protection over a 6-year period. Improved health-related quality of life	Off-target gene silencing, inflammation, toxicity, viral or bacterial infection, nasopharyngitis, abnormal blood clotting, gallbladder disease symptoms, elevated transaminases, nausea, injection site reactions, rash, fatigue, elevated transaminases, renal toxicity	[6,126,127]
mRNA vaccines	Pfizer-BioNTech BNT162b2, Moderna mRNA-1273	COVID-19	Intramuscular injection	41–95% effectiveness against infection	Possible pro-carcinogenic effects, injection-site reactions fatigue, headache, myalgia, fever	[6,110,112,121,128,129,130,131]

**Table 2:** Key characteristics of the considered antiviral and anticancer RNA-based therapeutic applications.

RNA Type	Drug Name	Target	Modification and/or Delivery System	Disease Indications	Clinical Trial Phase, Status	Clinical Trials.gov Identifier	Ref.
**miRNA**	Miravirsen	miR-122	LNA-modification	Chronic Hepatitis C	I, completed, II, completed	NCT01200420, NCT01646489, NCT00688012, NCT00979927	[132]
	RG-101 (Temavirsen)	miR-122	GalNAc conjugate	Chronic Hepatitis C	II, completed	EudraCT Number: 2015-004702-42	[133]
	MesomiR-1 (TargomiRs)	miR-16 (mimic)	EnGeneIC delivery platform	Malignant pleural mesothelioma, non-small cell lung cancer	I, completed	NCT02369198	[134]
	MRX34	miR-34 (mimic)	Liposomal	Primary liver cancer, hematologic malignancies, melanoma	I, terminated, I/II, withdrawn	NCT01829971, NCT02862145	[135]
	MRG-106 (cobomarsen)	miR-155 inhibitor	LNA-modification	MF, CLL, diffuse large B-cell lymphoma (DLBCL), CTCL	I, completed, II, terminated	NCT02580552, NCT03713320, NCT03837457	[78]
	TTX-MC138	miR-10b inhibitor	TTX delivery platform (iron oxide nanocarrier)	Advanced solid tumour	Early I, completed, I/II, active, not recruiting	NCT05908773, NCT06260774	[136]
**siRNA**	ARO-HBV (JNJ-3989)	HBV RNAs	GalNAc conjugate	Hepatitis B, D	I, II, completed, II, terminated	NCT05275023, NCT05123599, NCT04667104, NCT04535544, etc.	[137]
	AB-729 (Imdusiran)	Viral antigens	GalNAc conjugate	Hepatitis B, D	II, completed, terminated, recruiting	NCT04980482, NCT06154278, NCT06277037, etc.	[138]
	ARC-520	HBV RNAs	Nanoparticle	Hepatitis B	I, completed, II, terminated, withdrawn	NCT02535416, NCT01872065, NCT02738008, etc.	[19]
	ALN-HBV02, VIR-2218 (elebsiran)	HBV RNA	2′-OME, 2′-F, GalNAc conjugate	Hepatitis B	I, II completed, active, III, active	NCT05612581, NCT05844228, NCT05970289, etc.	[139]
	Cotsiranib (STP-705)	TGF-β1, COX-2	polypeptide nanoparticle (PNP)	Basal Cell Carcinoma (BCC)	I, completed, II, completed	NCT04669808, NCT04293679, NCT04676633, NCT04844983, etc.	[140]
	STP-707	TGF-β1, COX-2	PNP	Solid tumour	I, completed, active	NCT05037149, NCT05309915	[141]
**ASO**	Fomivirsen (Vitravene)	major immediate early region 2 (IE2) of human CMV	No	Cytomegalovirus Retinitis	II, completed	NCT00002356, NCT00002156	[142]
	Bepirovirsen (GSK-3228836)	HBV RNA	2′-MOE	Hepatitis B	I, completed, II, completed	NCT06058390, NCT04971928, NCT04676724, etc.	[143]
	IGV-001	IGF type 1 receptor	Goldspire™	Glioblastoma	II, active	NCT04485949	[144]
	Imetelstat (RYTELO, GRN163L)	hTERT	Lipid conjugate	Solid tumour malignancies, lung cancer, Multiple myeloma, NSCLC, Breast Cancer, Lymphoma, Myelofibrosis, Thyroid cancer	I, II completed, terminated, active, III, terminated, active, FDA approved for Myelodysplastic Syndromes	NCT01256762, NCT01243073, NCT01242930, etc.	[145]
	Danvatirsen (AZD9150)	STAT3	ethyl (cEt) modifications	AML, NSCLC, NHL, DLBCL, Advanced Solid Tumours	I, completed, active, II, completed, terminated, active	NCT03421353, NCT03394144, NCT01839604, etc.	[146]
	Oblimersen (G3139, Genasense)	BCL2	PS	Melanoma, Adult Acute Myeloid Leukaemia (AML), Multiple Myeloma, Plasma Cell Neoplasm, Leukaemia	I, completed, terminated, active, II, completed, terminated, III, completed, terminated	NCT00543231, NCT00542893, NCT00518895, etc.	[147]
	Custirsen (OGX-011)	Clusterin	PS, 2′-MOE	Breast Cancer, Prostate Cancer, Non-small Cell Lung Cancer	I, II, III, completed	NCT01578655, NCT01188187, NCT00327340, NCT00258375, etc.	[148]
	Trabedersen (AP-12009, OT-101)	TGF-β2	CED—convection-enhanced delivery	NSCLC, Pancreatic Neoplasms, Melanoma, Colorectal Neoplasms, Glioblastoma, Anaplastic Astrocytoma	I, II, completed, active	NCT06579196, NCT00844064, NCT00431561	[149]
	BP1001	GRB2	Liposomal	AML, CML, solid tumours	I, completed, active, II, recruiting	NCT04196257, NCT02923986, NCT02781883, NCT01159028	[150]
	LY2181308	Survivin	-	AML, prostate cancer, NSCLC	II, completed	NCT01107444, NCT00642018, NCT00620321, NCT00415155	[151]
	Apatorsen (OGX-427)	Hsp27	2′-methoxyethyl-modified	Prostate, breast, bladder cancer, NSCLC	I, II, completed	NCT01844817, NCT01829113, NCT01780545, NCT01454089, etc.	[152]
	SD-101	TLR 9	No	Melanoma, HNSCC, pancreatic adenocarcinoma, NSCLC, Lymphomas, Prostatic Neoplasms, Chronic Hepatitis C (NCT00823862)	I, II, completed	NCT04050085, NCT03831295, NCT03410901, NCT03322384, etc.	[153]
	EZN-2968	HIF-1α	LNA	Advanced solid tumours, lymphoma	I, completed	NCT02564614, NCT01120288, NCT00466583	[154]
	AZD4785	KRAS	ethyl (cEt) residues	NSCLC, advanced solid tumours	I, completed	NCT03101839	[155]
	GTI-2040	RNR R2	PS	Leukaemia (AML, CML), advanced solid tumours, NSCLC	I, II, completed	NCT00565058, NCT00459212, NCT00087165, NCT00084643, etc.	[156]
**Self-amplifying RNA (saRNA) vaccine**	ARCT-154		LNP	COVID-19, influenza	I, II, III completed	NCT06279871, NCT05037097, NCT05012943	[157]
**Small Activating RNA (saRNA)**	MTL-CEBPA	CEBPA	liposome encapsulated	Advanced Hepatocellular Carcinoma, Solid Tumour, Liver Cancer, Hepatitis B/C	I, II completed, active	NCT05097911, NCT04710641, NCT04105335, NCT02716012	[158]
	RAG-01	CDKN1A (p21 gene)	LiCO™ delivery technology	Non-Muscle-Invasive Bladder Cancer (NMIBC)	I, recruiting	NCT06351904	[159]
**circRNA**	ORN252			Oncology and autoimmune applications	I, recruiting	NCT07439796	[160]
**mRNA**	mRNA-1345	encoding the RSV fusion (F) glycoprotein	LNP	Respiratory syncytial virus (RSV) infection	I, II, III, completed, active	NCT07117487, NCT06143046, NCT06097299, NCT06067230, etc.	[161]
	mRNA-1647	HCMVgB and pentameric gH/gL/UL128/UL130/UL131A glycoprotein complex	LNP	Cytomegalovirus infection	I, II, completed	NCT05397223, NCT05105048, NCT04975893, NCT04232280, NCT03382405, NCT01195571	[162]
	mRNA-1944	encoded for functional antibody CHKV-24	LNP	Chikungunya Virus	I, completed	NCT03829384	[163]
	mRNA-4157 (V-940)	patient-specific tumour neoantigens	LNP	NSCLC, Melanoma	I, II, III, completed, active	NCT07221474, NCT06961006, NCT06833073, NCT06623422, etc.	[164]
	Autogene Cevumeran (BNT-122)	unspecified TAAs	LNP	Colorectal, Pancreatic ductal adenocarcinoma (PDAC)	I, II, completed, active	NCT06534983, NCT05968326, NCT03815058, NCT03289962	[165]

## Data Availability

The original contributions presented in this study are included in the article. Further inquiries can be directed to the corresponding author.
